# ECG Features of Pulmonary Embolism in a Patient With Normal D-Dimer and Hypoxia

**DOI:** 10.7759/cureus.49433

**Published:** 2023-11-26

**Authors:** Mehul S Amin, Rifat Ershad, Nikhil Kadam, Zahid Khan

**Affiliations:** 1 Internal Medicine, Southend University Hospital, London, GBR; 2 General Medicine, Basildon Hospital, London, GBR; 3 Internal Medicine, Mid and South Essex NHS Foundation Trust, Southend on Sea, GBR; 4 Acute Medicine, Mid and South Essex NHS Foundation Trust, Southend on Sea, GBR; 5 Cardiology, Bart’s Heart Centre, London, GBR; 6 Cardiology and General Medicine, Barking, Havering and Redbridge University Hospitals NHS Trust, London, GBR; 7 Cardiology, Royal Free Hospital, London, GBR

**Keywords:** therapeutic anticoagulation, ct pulmonary angiogram (ctpa), ecg abnormalities, reliability of plasma d-dimer, pulmonary embolus

## Abstract

Pulmonary embolism is a life-threatening condition that requires urgent treatment. We present the case of a 76-year-old male referred to our medical team with dyspnoea, shortness of breath on exertion, and chest pain. Upon further questioning, the patient reported a two-week history of right-sided parasternal pleuritic chest pain without radiation. He denied any history of haemoptysis, calf swelling or pain, recent surgery, and reduced mobility. The patient had a medical history of bilateral cataracts, glaucoma, and hypertension. Clinical examination was unremarkable except for requiring 2L/minute supplemental oxygen to maintain an oxygen saturation of 94%, and blood tests were unremarkable, including a normal D-dimer. Chest radiography revealed no obvious pathological findings. However, the electrocardiogram showed a right bundle branch, sinus tachycardia, and an S1Q3T3 pattern. A computed tomography pulmonary angiogram confirmed pulmonary emboli within the right lower lobe segmental artery, extending into the bilateral basal segmental branch and posterior basal segmental branch. The patient was commenced on low molecular weight heparin initially followed by rivaroxaban 20 mg once daily. This case highlights the importance of having a high degree of suspicion for pulmonary embolism, and D-dimer is an important screening test that can be normal.

## Introduction

Pulmonary embolism (PE) is a potentially life-threatening condition that occurs when a thrombus occludes the pulmonary arteries, which can impair blood flow and oxygenation of haemoglobin. Pulmonary emboli can originate from the deep venous system of the legs (deep vein thrombosis), which can travel to the pulmonary arteries. Pulmonary embolism is associated with high mortality and morbidity rates. The clinical presentation of PE can be highly variable, which poses a substantial diagnostic challenge [1-2].

The gold standard for the investigation of pulmonary embolism is computed tomography pulmonary angiogram (CTPA), but to reduce the overexposure of radiation patients, D-dimer levels and clinical scoring systems are used to assess whether patients are at high and low risk of having a pulmonary embolism [3].

Elevated D-dimer levels can be indicative of fibrinolysis from thrombosis; they serve as a useful screening tool for PE because of their high sensitivity. However, it has a very low specificity for PE. Hence, current guidelines in the United Kingdom (UK) recommend the use of clinical prediction scores, such as Well’s score, in conjunction with D-dimer testing to stratify the pretest probability of PE. Well’s score incorporates factors, such as clinical signs of deep vein thrombosis, alternative diagnoses being less likely, heart rate, immobilization, previous history of deep vein thrombosis or pulmonary embolism, haemoptysis, and malignancy. These factors put patients into low or high pretest probability categories, thereby guiding further diagnostic strategies, including D-dimer use [4]. However, cases of pulmonary embolisms with normal D-dimer levels, including age-adjusted D-dimer calculations, have still been reported, highlighting the importance of high-degree suspicion due to false negative values requiring careful assessment of additional investigations such as electrocardiogram changes [5-7].

Electrocardiography (ECG) is a simple and inexpensive investigation that is readily available; however, the findings are often non-specific to the presentation of pulmonary embolism. There have been multiple findings associated with pulmonary embolism, including right strain pattern, right bundle branch (either complete or partial), axis deviation, S1Q3T3 pattern, T wave inversion in anterior pectoral leads, tachycardia, atrial fibrillation, and ST elevation in the aVR lead [8-11]. The most common findings associated with pulmonary embolism include sinus tachycardia and S1Q3T3 pattern followed by the other ECG changes mentioned above in one of the studies [11]. Studies have reported the use of various ECG criteria in the context of acute PE, including the Daniel Score for cardiac stress from acute pulmonary embolism, which has been investigated but not widely adopted [11].

Here, we present the case of a 76-year-old male with clinical symptoms suggestive of PE, but with a normal D-dimer level. This highlights the complexities of diagnosing PE and prompts a re-evaluation of diagnostic strategies in atypical cases.

## Case presentation

We present the case of a 76-year-old male who presented to our hospital with a two-week history of non-radiating right-sided chest pain. Upon further questioning, he reported pleuritic chest pain described as sharp, worsening on deep inspiration with no alleviating factors, and scored 6 out of 10 on a pain scale. He also reported dyspnoea and shortness of breath on exertion. He denied haemoptysis, pyrexia, infective symptoms, weight loss, calf pain, prolonged immobility, recent long-haul flights, history of malignancy or recent surgery. Past medical history was significant for hypertension, bilateral cataracts, and glaucoma. His regular medications included latanoprost eye drops in both eyes, irbesartan 300 mg once daily (OD), azarga drops in both eyes bis in die (BD), travoprost one drop in both eyes OD, sertraline 50 mg OD, atenolol 75 mg OD, amlodipine 10 mg OD, and bendroflumethiazide 2.5 mg OD. There was no family history of deep vein thrombosis or pulmonary embolism. He was independent of activities of daily living and mobility, was an ex-smoker with a 15-pack-year history and denied any alcohol use.

**Figure 1:**
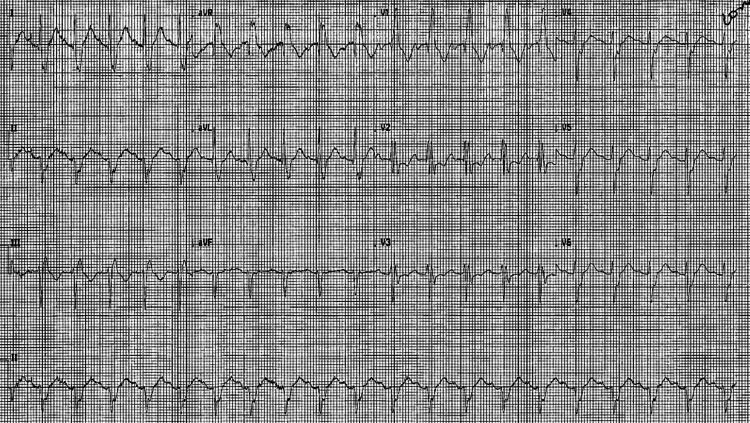
Electrocardiography shows sinus tachycardia, left axis deviation (positive QRS in lead 1), right bundle branch block (V1-V2) and S1Q3T3

The Initial assessment revealed that the patient was comfortable at rest, with no cyanosis or respiratory distress. Clinical examination showed a National Early Warning Score (NEWS2) score of 4, the airway was patent, and the trachea was central. The chest was clear on auscultation and resonant with percussion. The oxygen saturation on pulse oximetry was 94% with 2 L/minute of supplementary oxygen, and the respiratory rate was 18. Heart sounds were normal, pulse was regular at 108 bpm, capillary refill was 3 s centrally, and blood pressure was 190/115 mmHg. Glasgow coma scale score was 15, blood glucose level was 9.1, and temperature was 36.7C. The remaining physical examination results were unremarkable. The patient’s Coronavirus Disease 2019 polymerase chain reaction test results were negative. The laboratory values and arterial blood gas results are presented in Table 1 and Table 2, respectively. Electrocardiography revealed normal sinus tachycardia, left axis deviation, right bundle branch block and S1Q3T3 (Figure 1).

**Table 1:** Shows the initial blood test results for the patient on admission

Blood Test	Patient value	Normal Range
Haemoglobin	147 g/L	133 - 173 g/L
White cell count	9.3 * 10^9^/L	3.8 - 11 * 10^9^/L
Platelet count	205 * 10^9^/L	150 – 400 * 10^9^/L
C reactive Protein	1 mg/L	0 - 5 mg/L
Prothrombin time	12.1 seconds	10.3 - 13.3 seconds
Activated partial thromboplastin time	31.9 seconds	25.7 - 35.3 seconds
INR	1.0	0.8-1.2
D-dimer	168	<243 ng/L
Trop	37 and 36	<14ng/L
Sodium	139 mmol/L	133 - 146 mmol/L
Potassium	mmol/L	3.5 - 5.3 mmol/L
Urea	4.8 mmol/L	2.5 -7.8 mmol/L
Creatinine	94 umol/L	59 - 104 umol/L

**Table 2:** Shows arterial blood glass on 2 litres of supplementary oxygen

	Patient values	Normal Range
pH	7.43	7.35 - 7.45
pCO2	4.23	4.6 - 6 kPa
pO2	9.54	10.7 - 13.3 kPa
Bicarbonate	29.6 mmol/L	22-26 mmol/L
Glucose	9.1 mmol/L	4.4- 6.1 mmol/L
Lactate	1.4 mmol/L	0.4 - 2.2 mmol/L

**Figure 2:**
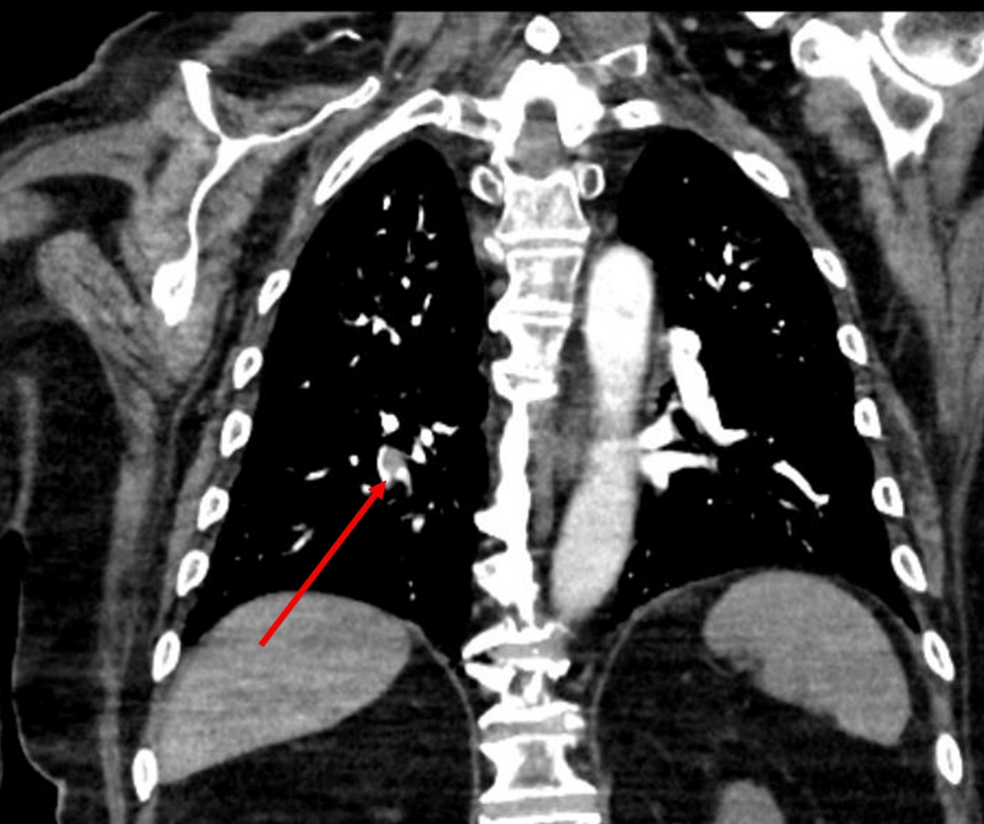
Computerized tomography pulmonary angiogram shows right-sided lower lobe pulmonary emboli as demonstrated by the red arrow

Chest radiography findings were normal, and the patient did not show any signs of infection. In the absence of any convincing signs of infection, oxygen requirement and ECG changes, the patient under CTPA demonstrated pulmonary emboli within the right lower lobe segmental artery, extending into the bilateral basal segmental branch and posterior basal segmental branch (Figure 2). There were no features of right-sided heart strain. He received a treatment dose of enoxaparin at 1.5 mg/kg dose of 160 mg enoxaparin once daily. Echocardiography was performed the following day to assess right heart pressures and to rule out any cardiac cause for pulmonary embolism, which showed normal left ventricular function with an ejection fraction of 55% and no evidence of right heart strain.

He was successfully discharged with rivaroxaban 20 mg once daily on Day 5 and was referred to the respiratory outpatient clinic for follow-up. He was reviewed in the respiratory outpatient clinic and advised lifelong anticoagulation with rivaroxaban for an unprovoked pulmonary embolism following a computerized tomography scan of the abdomen and pelvis, excluding malignancy.

## Discussion

This clinical scenario prompts a closer examination of the difficulties surrounding pulmonary embolism diagnosis. The causes of pulmonary embolism can be broadly split into two categories, provoked and unprovoked. Provoked pulmonary embolism refers to cases in which there is a known specific risk factor or precipitant causing an embolism. Risk factors include recent surgery, trauma, prolonged immobilization, active cancer, coagulation disorders, drugs such as combined oral contraceptive pills, and pregnancy. In unprovoked cases, no identifiable cause or precipitant led to the formation of a pulmonary embolus, as in the case described above.

The presentation of symptoms over two weeks with shortness of breath and pleuritic chest pain in this case, with normal biochemistry and initial imaging, warranted further assessment. In the absence of any major risk factors, the suspicion of pulmonary embolism was not initially high in the emergency department of a low-risk patient. The initial investigations did not yield a definitive diagnosis. Therefore, D-dimer levels returned to normal. This was compounded by the fact that the patient's presentation did not yield a sufficiently high Well’s score for CTPA to be carried out as the first-line investigation. However, an abnormal electrocardiogram and oxygen requirement warrant further investigation to confirm pulmonary embolism.

Electrocardiograms are helpful for assessing acute coronary syndromes in patients with chest pain. However, a wide range of changes have been reported in the context of pulmonary embolism. The most common finding is sinus tachycardia; however, additional features such as right strain pattern, right bundle branch (either complete or partial), axis deviation, S1Q3T3 pattern, T-wave inversion in anterior pectoral leads, ST segment changes and atrial arrhythmias have been recorded [8-11]. Changes in the electrocardiogram in the context of cardiac stress from acute pulmonary embolism have been investigated, but not widely adopted from Daniel’s score [11-13]. ECG changes when coupled with oxygen requirement, negative D-dimer test, normal chest radiograph and unremarkable examination raise the possibility of a pulmonary embolism due to high mortality and morbidity associated with the condition.

A systematic review reported a 0.97 sensitivity and specificity of 0.41 for d-dimer while CTPA had a sensitivity of 0.94 and specificity of 0.98 in the assessment of pulmonary embolism [12]. D-dimer is a useful marker for ruling out PE if the Well’s score is below 4; however, this case presents a clinical dilemma in which a normal D-dimer level and the use of Well’s score for PE are underestimated. In such cases, a high degree of suspicion is required for pulmonary embolism due to its vague presentations such as breathlessness, syncope, and chest pain. A negative D-dimer alone without imaging in cases of suspected pulmonary embolism warrants further assessment because of high mortality and morbidity [5]. The higher sensitivity and specificity make CTPA the investigation of choice for pulmonary embolism diagnosis. In cases where CTPA is not readily available, guidelines recommend starting treatment while awaiting imaging in suspected cases.

## Conclusions

Considering the widespread use of D-dimer as a screening tool for the exclusion of pulmonary embolism, it further highlights the importance of high clinical suspicion, and as evidenced by this case, the sensitivity of the test is limited. Additionally, the presence of oxygen requirement, electrocardiogram changes and normal initial investigations should raise the possibility of pulmonary embolism. Thus, in such cases, we should consider gold standard investigations, such as CTPA, and counselling patients on the risk of radiation due to its high sensitivity. In scenarios where CT is not readily available, there is a high degree of suspicion of pulmonary embolism despite negative D-dimer, prompt treatment should be commenced.
